# Using Fourier ptychography microscopy to achieve high-resolution chromosome imaging: an initial evaluation

**DOI:** 10.1117/1.JBO.27.1.016504

**Published:** 2022-01-31

**Authors:** Ke Zhang, Xianglan Lu, Xuxin Chen, Roy Zhang, Kar-Ming Fung, Hong Liu, Bin Zheng, Shibo Li, Yuchen Qiu

**Affiliations:** a University of Oklahoma, Stephenson School of Biomedical Engineering, Norman, Oklahoma, United States; b University of Oklahoma, School of Electrical and Computer Engineering, Norman, Oklahoma, United States; c University of Oklahoma Health Sciences Center, Department of Pediatrics, Norman, Oklahoma City, Oklahoma, United States; d University of Oklahoma Health Sciences Center, Department of Pathology, Norman, Oklahoma City, Oklahoma, United States

**Keywords:** Fourier ptychography microscopy, analyzable metaphase chromosome, leukemia diagnosis, high throughput scanning

## Abstract

**Significance:**

Searching analyzable metaphase chromosomes is a critical step for the diagnosis and treatment of leukemia patients, and the searching efficiency is limited by the difficulty that the conventional microscopic systems have in simultaneously achieving high resolution and a large field of view (FOV). However, this challenge can be addressed by Fourier ptychography microscopy (FPM) technology.

**Aim:**

The purpose of this study is to investigate the feasibility of utilizing FPM to reconstruct high-resolution chromosome images.

**Approach:**

An experimental FPM prototype, which was equipped with 4×/0.1  NA or 10×/0.25  NA objective lenses to achieve a theoretical equivalent NA of 0.48 and 0.63, respectively, was developed. Under these configurations, we first generated the system modulation transfer function (MTF) curves to assess the resolving power. Next, a group of analyzable metaphase chromosomes were imaged by the FPM system, which were acquired from the peripheral blood samples of the leukemia patients. The chromosome feature qualities were evaluated and compared with the results accomplished by the corresponding conventional microscopes.

**Results:**

The MTF curve results indicate that the resolving power of the 4×/0.1  NA FPM system is equivalent and comparable to the 20×/0.4  NA conventional microscope, whereas the performance of the 10×/0.25  NA FPM system is close to the 60×/0.95  NA conventional microscope. When imaging the chromosomes, the feature qualities of the 4×/0.1  NA FPM system are comparable to the results under the conventional 20×/0.4  NA lens, whereas the feature qualities of the 10×/0.25  NA FPM system are better than the conventional 60×/0.95  NA lens and comparable to the conventional 100×/1.25  NA lens.

**Conclusions:**

This study initially verified that it is feasible to utilize FPM to develop a high-resolution and wide-field chromosome sample scanner.

## 1 Introduction

Leukemia is one category of blood cancers that commonly occur in both female and male patients.^1^ For the diagnosis and treatment of leukemia, chromosome karyotyping^2^ is critically important; it organizes and pairs all chromosomes in order of decreasing length. In this process, cytogeneticists have to search the whole specimen slide and identify the analyzable metaphase chromosomes, which is a time-intensive and tedious operation. To reduce the clinician’s workload and enhance the diagnostic efficiency, many research studies have been focused on the development of automated slide scanners^3^ and the associated computer-aided detection schemes,^4^^,^^5^ aiming to accomplish quick cell digitization and identification. Despite some encouraging progress, the performance of the current scanners is majorly impeded by the long scanning time, which can be attributed to the limited space-bandwidth product of the objective lens.^6^^,^^7^ With few high-cost exceptions,^8^ this limitation indicates that it is very difficult for the objective lens to simultaneously accomplish a large field of view (FOV) and high spatial resolution, which implies that high-resolution objective lenses will have a much smaller FOV than the low-resolution lens. Considering that the chromosome images must be obtained with high spatial resolution to ensure enough details for clinical karyotyping, the corresponding high-magnification scanning will be significantly slower as each acquisition only covers a much smaller area of the specimen.

Meanwhile, the recent technology of Fourier ptychography microscopy (FPM)^9^^,^^10^ is an emerging strategy to address the challenge of limited space-bandwidth products. This method first collects a number of low-magnification images under different sample illumination conditions and then synthesizes them together to reconstruct a high-resolution image. Since the data acquisition is under low magnification, it inherits the advantage of low-resolution scanning systems, namely, the large FOV and large depth of field (DOF).^11^ For example, in current clinical practice, the 100×/1.25  NA objective lens is used to ensure the band pattern sharpness. Given the typical objective lens with an optical field number (OFN) of 22, the corresponding FOV is ∼0.22  mm. Meanwhile, if FPM is utilized to achieve the same band pattern quality under the 10×/0.25  NA lens, the FOV of the objective lens (with the same OFN number) is enhanced to 2.2 mm; thus the scanning efficiency can be greatly enhanced. Moreover, due to the large system DOF, FPM can also vastly reduce the precision requirement of the moving stage, and the total cost of the scanner can be significantly reduced. Although this method has been applied in many different clinical scenarios, no previous research has focused on investigating the feasibility of utilizing FPM on high-resolution metaphase chromosome imaging.

In this study, we comprehensively assess the performance of an FPM system and its clinical utility on chromosome imaging. The modulation transfer function (MTF) curves are first estimated, aiming to assess the system resolving power. Next, the analyzable metaphase chromosomes acquired from the peripheral blood samples of leukemia patients are imaged by FPM system, and the chromosome feature qualities are compared with the results captured by the corresponding conventional microscopes. All experimental details are presented in the following sections.

## 2 Materials and Methods

### 2.1 Experimental Set-Up

In this experiment, a Fourier ptychography-based microscope was developed on the basis of the Thorlabs Cerna Microscope platform (Thorlabs, New Jersey, USA), which is equipped with either 4×/0.1  NA or 10×/0.25  NA Olympus objective lenses (Olympus, Tokyo, Japan) and a FL20BW CMOS camera (Tuscen, Fuzhou, China). The focal length of the platform tube lens is 150 mm; thus the actual magnification is ∼3.3× and 8.3× for the 4×/0.1  NA and 10×/0.25  NA objective lenses, respectively, as 180 mm is the standard focal length for the Olympus infinity corrected optical system. To accomplish the tilted illumination, a 32×32 LED panel (Adafruit, New York, USA) was placed under the sample stage, with the vertical stage-sample distance being ∼70  mm. During the image acquisition, the central 225 (15×15) green (4 mm spacing, central wavelength 0.53  μm) LEDs were used, which yields an illumination NA of ∼0.38. Therefore, the equivalent system ${\mathrm{NA}}_{\mathrm{eq}}$ is ∼0.48 and ∼0.63 for the 4× and 10× objective lens configurations, respectively. Our FPM system can also be performed as a regular microscope for 20× imaging (0.40 NA). Meanwhile, the high-magnification comparative imaging was conducted on a Nikon Ni transmissive microscope (Nikon, Tokyo, Japan), which is equipped with 60×/0.95  NA or 100×/1.25  NA objective lenses.

### 2.2 Fourier Ptychography Theory and Image Reconstruction

**Fig. 1:**
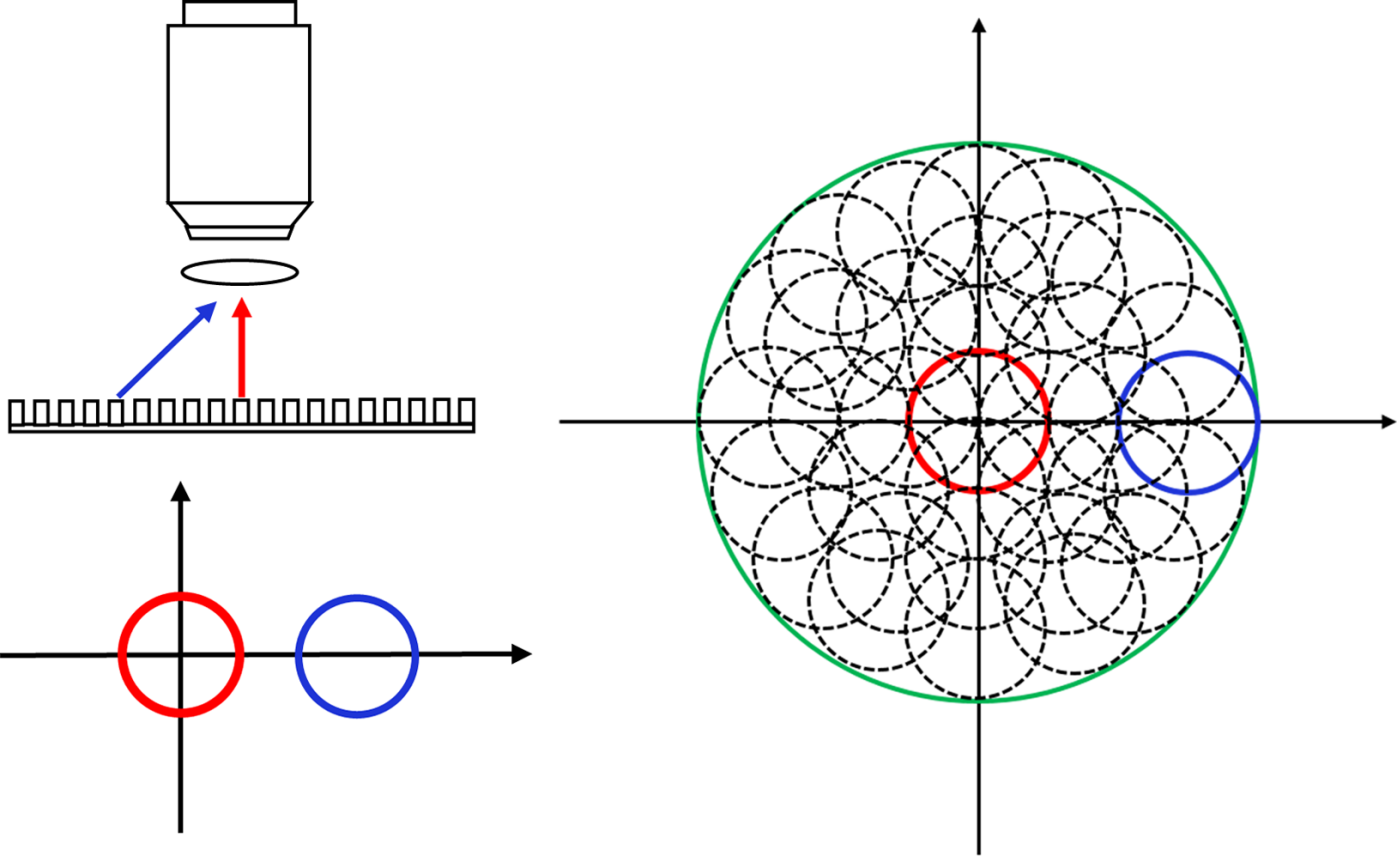
The principle of FPM technology.

FPM is essentially one kind of aperture synthesis technology.^12^^,^^13^ From the perspective of frequency domain, the objective lens is a low-pass circular filter. In regular imaging, the sample s(x) is illuminated along the perpendicular angle of incidence (Fig. 1, red). The objective lens works as a circular shape low-pass filter on the corresponding spectrum (i.e., its Fourier transform) S(k), and the filter cutoff frequency is estimated as 2πNA/λ (λ is the illumination wavelength).^14^ However, if the (partially) coherent light illuminates the sample with an angle of incidence $\theta_{x}$ in the X direction (Fig. 1, blue), the corresponding spectrum will be shifted as $S(k-k_{x})$, $k_{x}=2\pi \;\sin\;\theta_{x}/\lambda$, and the objective lens will then filter the spectrum and collect the information within the same circle area, but the center is changed to $k-k_{x}$. Therefore, if the sample is illuminated under a sequence of different angles of incidence, the system can collect the frequency information from different areas of the corresponding sample spectrum. After we computationally synthesize this information together, the cutoff frequency can be extended to $2\pi (\sin\;\theta_{\max}+\mathrm{NA})/\lambda$, with $\theta_{\max}$ being the maximal angle of incidence (Fig. 1, green circle). The resolving power will then be vastly improved.

Accordingly, reconstructing the high-resolution image from the acquired raw patterns is equivalent to reconstructing the corresponding spectrum. Given that the complex spectrum requires both intensity and phase information and the raw patterns only record the sample intensity, the missing phase information (phase retrieval) needs to be restored for the spectrum reconstruction. Moreover, the algorithm has to estimate the optimal pupil function to minimize the aberration and enhance the final image quality. As a result, in this study, we adopt a phase restoration algorithm embedded with pupil function recovery,^15^^,^^16^ which iteratively updates the reconstructed spectrum until the preset accuracy tolerance is satisfied. For this method, we first divide the entire spectrum of the reconstructed image into N different subregions, each of which corresponds to one obtained raw pattern. For each iteration, the spectrum update is performed subregion by subregion, which consists of the following three steps. First, pupil function $P^{\left(i\right)}(k)$ is applied on the subregion spectrum $S_{m}^{i}(k-k_{m})$: $\Psi_{m}^{\left(i\right)}(k)=S_{m}^{\left(i\right)}(k-k_{m})P^{\left(i\right)}(k)$, where i denotes the i’th iteration and m denotes the m’th subregion. In addition, $k_{m}$ is defined as $\left(2\pi \;\sin\;\theta_{xm}/\lambda ,2\pi \;\sin\;\theta_{ym}/\lambda \right)$, and $\left(\theta_{xm},\theta_{ym}\right)$ are the X and Y direction illumination angles, respectively. The spatial representation $\psi_{m}^{\left(i\right)}(r)=F^{-1}[\Psi_{m}^{\left(i\right)}(k)]$ is also computed by inverse Fourier transform correspondingly. Next, the captured raw pattern $I_{p}(r)$ is applied on the spatial representation as the intensity constraint: $\phi_{m}^{\left(i\right)}(r)=\sqrt{I_{p}(r)}\frac{\psi_{m}^{\left(i\right)}(r)}{\left|\psi_{m}^{\left(i\right)}(r)\right|}$. Finally, using Gaussian–Newton’s method, the subregion spectrum $S_{m}^{i}(k-k_{m})$ and pupil function $P^{\left(i\right)}(k)$ are updated based on the constrained result $\Phi_{m}^{\left(i\right)}(k)=F^{-1}[\phi_{m}^{\left(i\right)}(r)]$.

### 2.3 Use Standard Resolution Target and Clinical Specimen to Evaluate the Resolving Power of FPM System

To assess the resolving power, the MTF curves of the FPM system were measured under two configurations (i.e., 4× and 10× raw image acquisition). As a comparison, we also measured the MTF curves of the conventional microscope equipped with 20×/0.4  NA, 60×/0.95  NA, and 100×/1.25  NA objective lenses. Currently, which approach should be the standard metric to assess the performance of a coherent system has not been determined as the object phase variation may enhance the system power of resolving two close points.^6^ Given that the standard resolution pattern does not contain any phase variance, our MTF-based performance assessment may actually underestimate the performance of the investigated FPM system. Meanwhile, it is widely accepted that the MTF curve can objectively evaluate (neither underestimate nor overestimate) the performance of incoherent imaging systems such as conventional microscopes. Therefore, when using the MTF curve, we would rather underestimate our FPM system than overestimate it, and the corresponding results of performance comparison still remain solid.

Accordingly, a high-resolution (up to 3649  lp/mm) USAF 1951 target (Newport, California, USA) was adopted, and the contrast of the imaged bar patterns was measured at a sequence of discrete frequencies from zero to the spatial resolution.^17^ During the measurement, the test target was placed on the sample stage and adjusted to the in-focused position. For each pattern on the captured target, the contrast was calculated by^18^ $C=\frac{I_{\max}-I_{\min}}{I_{\max}+I_{\min}}$, where $I_{\text{max}}$ and $I_{\text{min}}$ are the average maximal and minimal values of the bar patterns, respectively. Based on these estimated contrast values, one smooth normalized MTF curve was generated by the curve fitting algorithm.^19^

To evaluate its clinical utility, a certain number of analyzable metaphase chromosomes were also imaged by our FPM system. All of the cells were collected from the blood samples of leukemia patients and processed under standard clinical procedure in our medical center. The chromosomes were imaged by both the FPM system and the corresponding equivalent conventional microscopes (e.g., under 60×/0.95  NA and 100×/1.25  NA objective lenses). The chromosome band patterns depicted on each generated image were assessed and compared between these two groups of results (i.e., FPM versus conventional system). To evaluate the impact of the DOF on the feature qualities,^17^ each chromosome was imaged in a series of different positions, including the in-focused position and other positions outside the focal plane.

## 3 Results

**Fig. 2:**
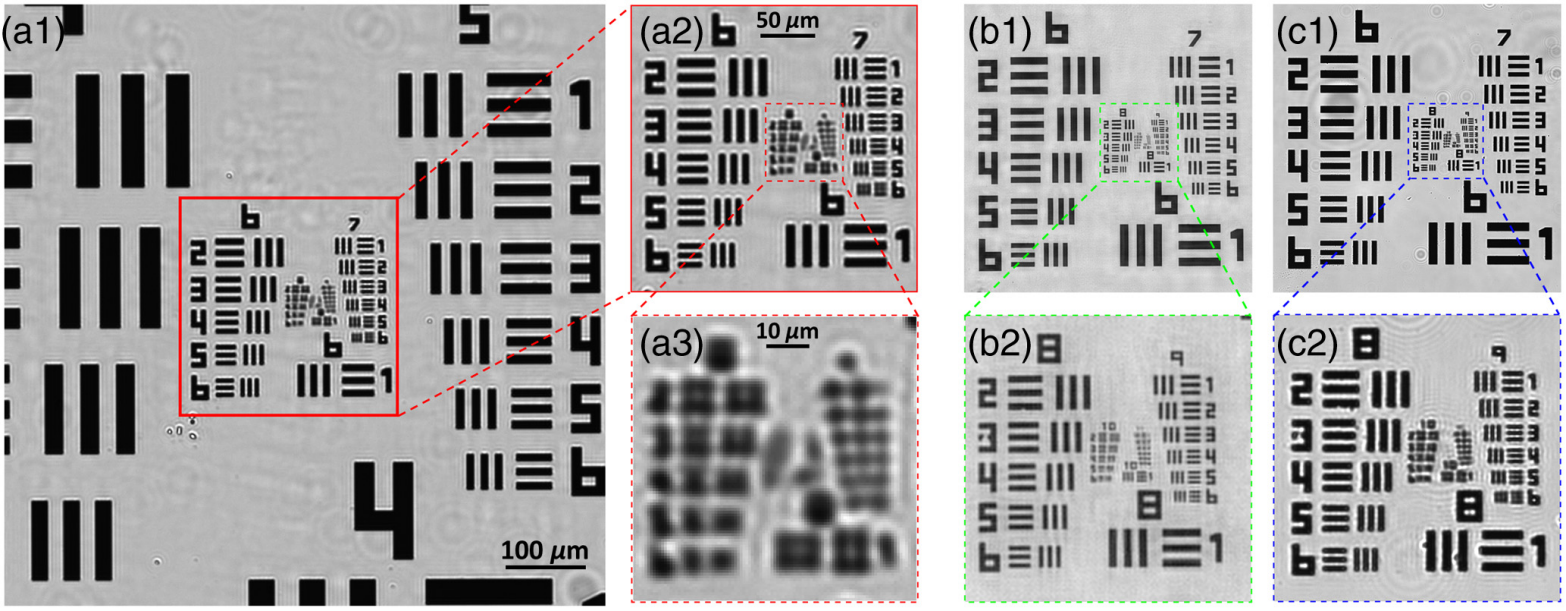
The images of a standard USAF 1951 resolution target, generated by (a) 4×/0.1  NA objective lens; (b) FPM technology with 4×/0.1  NA objective lens; and (c) 20×/0.4  NA objective lens.

**Fig. 3:**
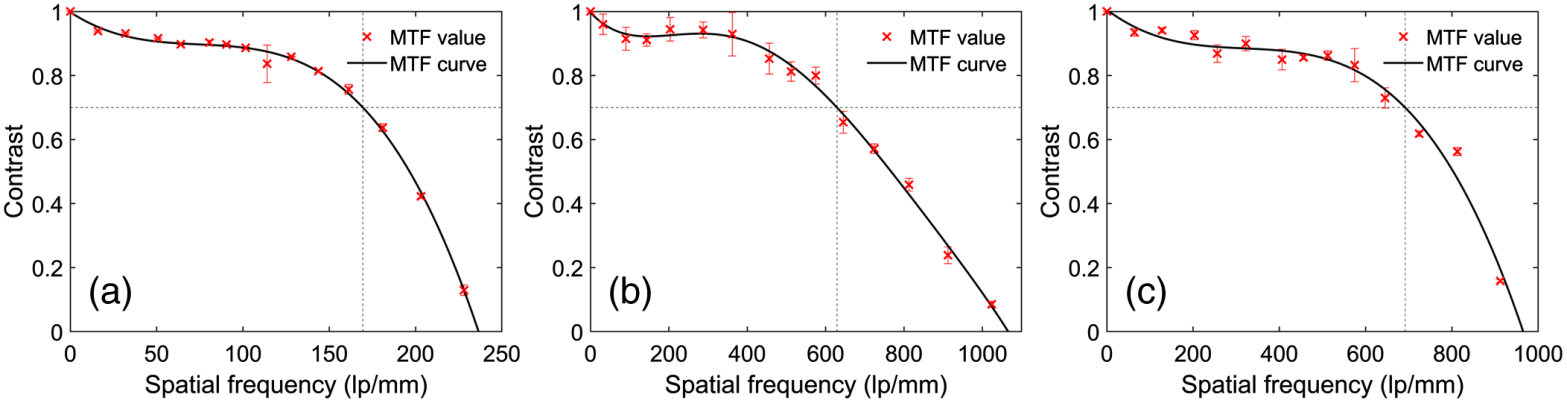
The measured MTF curves of different microscopes when using (a) 4×/0.1  NA objective lens; (b) FPM technology with 4×/0.1  NA objective lens; and (c) 20×/0.4  NA objective lens. Under these three imaging conditions, the cutoff frequencies are (a) 236.5  lp/mm, (b) 1065.83  lp/mm, and (c) 965.30  lp/mm, and the contrast values decrease to 0.7 at (a) 169.51  lp/mm, (b) 629.08  lp/mm, and (c) 691.58  lp/mm, respectively.

Figure 2 demonstrates the reconstructed images of a 1951 USAF resolution target. In Fig. 2(a), the sample is directly imaged under the 4×/0.1  NA objective lens, which is able to resolve the patterns up to group 7-3 (161  lp/mm). This result generally agrees with the theoretical computation (154  lp/mm). As indicated in Fig. 2(b), when using FPM with the 4×/0.1  NA objective, the spatial resolution of the reconstructed image increases to 813  lp/mm (group 9-5), which is equivalent to the result directly captured under the 20×/0.4  NA objective lens [Fig. 2(c)]. Meanwhile, Figs. 3(a)–3(c) demonstrate the estimated MTF curves when utilizing the 4×/0.1  NA objective lens, FPM technology, and 20×/0.4  NA objective lens, respectively. As predicted by the Fourier optics theory,^6^ the normalized contrast value monotonically decreases as the spatial frequency increases. The curve of the 4×/0.1  NA objective lens slowly decreases from 0 to 150  lp/mm and significantly decreases from 150  lp/mm to the cutoff frequency (∼237  lp/mm). Meanwhile, the contrast value of the FPM curve is above 0.7 when the frequency increases to 600  lp/mm, and the cutoff frequency is about 1066  lp/mm. The MTF curve of the 20×/0.4  NA objective lens is similar to the FPM results, as demonstrated in Fig. 3(c).

**Fig. 4:**
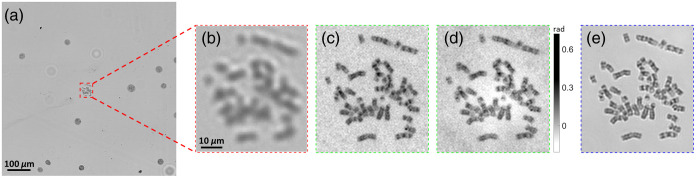
Sample images of one analyzable chromosome from the blood sample of leukemia patients, which are imaged using (a), (b) 4×/0.1  NA objective lens; (c), (d) FPM technology with 4×/0.1  NA objective lens; and (e) 20×/0.4  NA objective lens. (c) and (d) are the reconstructed amplitude and phase images, respectively.

Figure 4 demonstrates the microscopic images of one analyzable metaphase chromosome acquired from the blood samples of one leukemia patient. In clinical practice, the chromosome samples are stained by Giemsa dye to generate the “black and white” band (G-banding).^20^ Each chromosome has a unique band pattern as its own signature that is used for karyotyping or abnormality identification.^20^^,^^21^ The quality of the reconstructed images is largely determined from whether these band patterns are clearly resolved or not. Accordingly, when the cell is imaged under the 4×/0.1  NA objective lens [Fig. 4(a)], we can only distinguish this metaphase cell from other interphase cells with a circular shape but cannot recognize any details regarding the chromosome band patterns. Figures 4(c)/4(d) indicate the reconstructed amplitude and phase images by FPM equipped with the 4×/0.1  NA objective. Note that we rescaled the phase values to the range [0, 255] and then inverted the rescaled pixel values for figure demonstration because the raw phase image has a dark background (zero phase shift). A similar process was conducted on all of the following phase images. For the reconstructed amplitude/phase images [Figs. 4(c)/4(d)], some band patterns can be distinguished, and the band sharpness is comparable to the images captured by the 20×/0.4  NA objective lens [Fig. 4(e)].

**Fig. 5:**
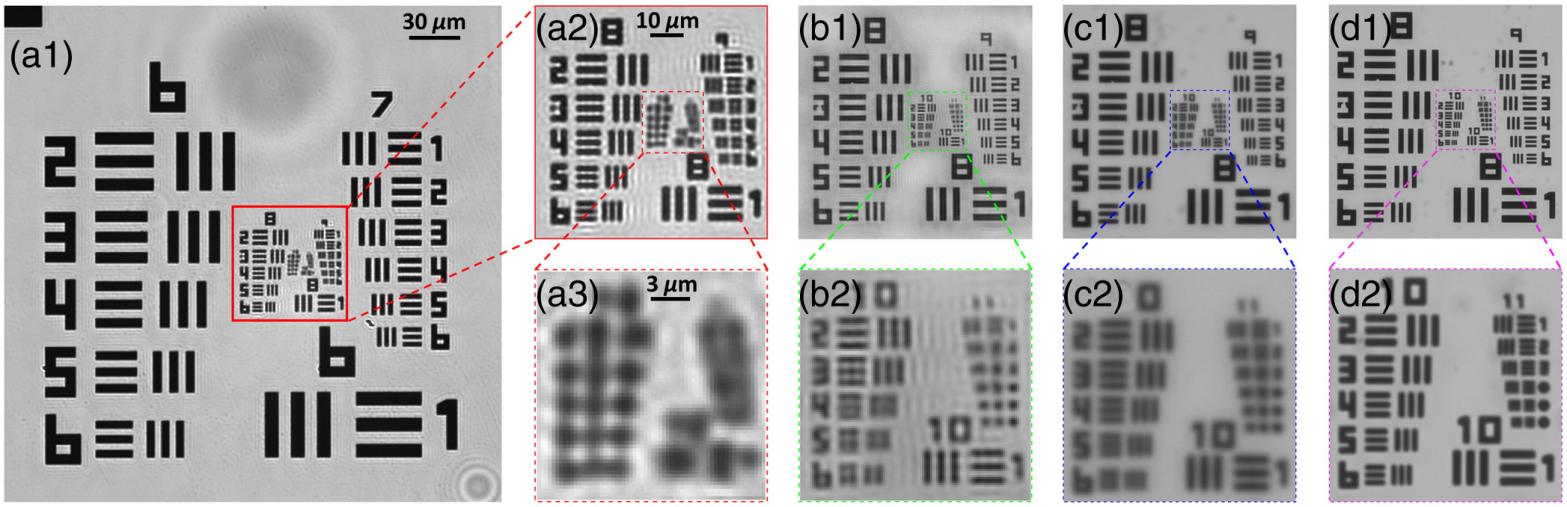
Images of the USAF1951 resolution target generated using (a) 10×/0.25  NA objective lens; (b) FPM technology with 10×/0.25  NA objective lens; (c) 60×/0.95  NA objective lens; and (d) 100×/1.25  NA objective lens.

**Fig. 6:**
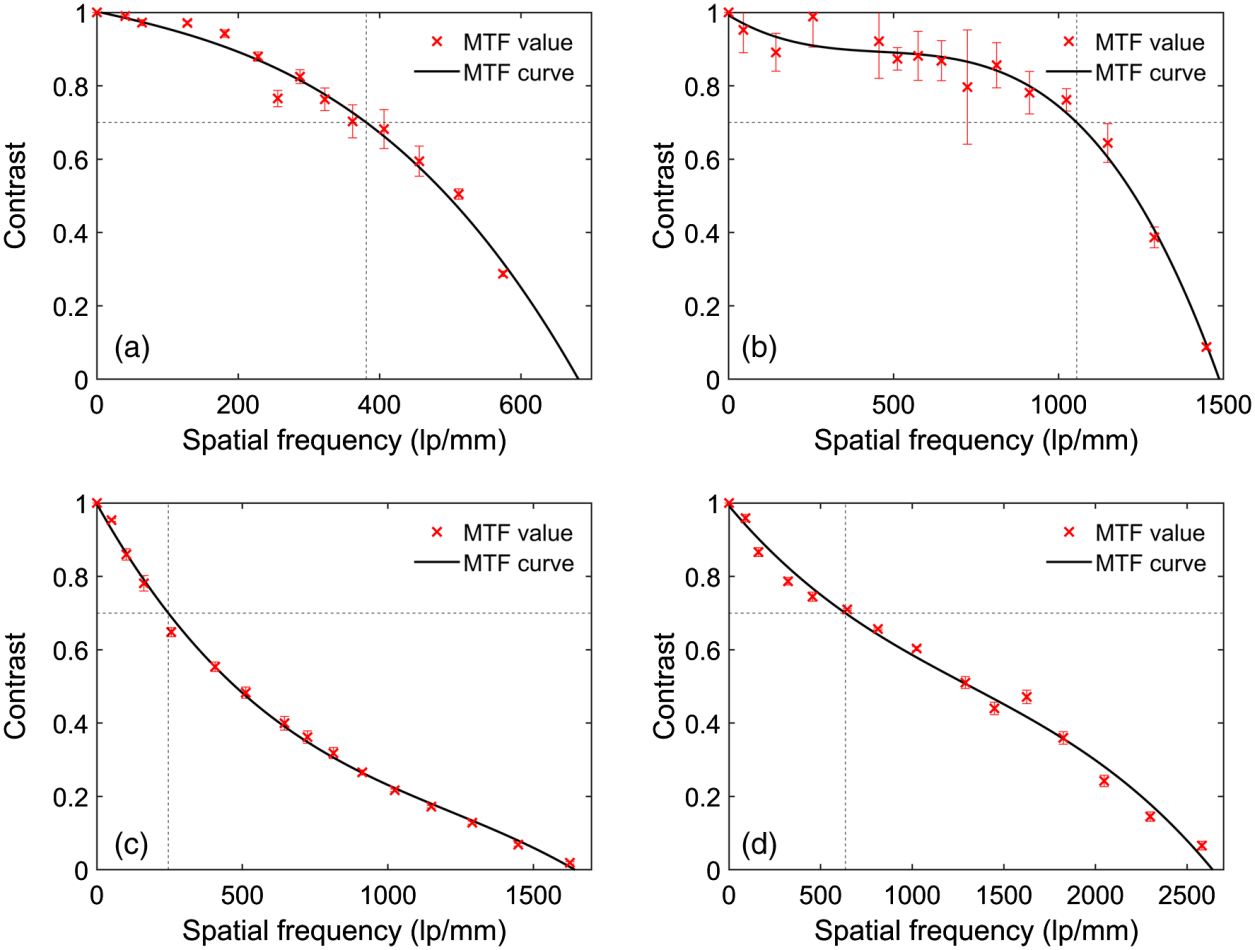
The measured MTF curves of different microscopes when using (a) 10×/0.25  NA objective lens; (b) FPM technology with 10×/0.25  NA objective lens; (c) 60×/0.95  NA objective lens; and (d) 100×/1.25  NA oil immersion objective lens. Under these four imaging conditions, the cutoff frequencies are (a) 681  lp/mm, (b) 1487  lp/mm, (c) 1642  lp/mm, and (d) 2639  lp/mm, and the contrast values decrease to 0.7 at (a) 381  lp/mm, (b) 1055  lp/mm, (c) 245  lp/mm, and (d) 635  lp/mm, respectively.

When the raw patterns were captured under the 10×/0.25  NA objective lens, the reconstructed USAF 1951 resolution target is indicated as Fig. 5. Figure 5(a) shows the target image that was directly captured by the 10×/0.25  NA objective lens; the minimum resolvable bar pattern is group 9-1 (512  lp/mm). When using the FPM technology [Fig. 5(b)], the spatial resolution vastly increases to 1290  lp/mm (group 10-3), which is comparable to the result of the 60×/0.95  NA objective lens [Fig. 5(c)]. Under the 100×/1.25  NA objective lens [Fig. 5(d)], the target image resolves the pattern bar up to group 10-6 (1825  lp/mm), which is significantly higher than the FPM results. Meanwhile, the corresponding MTF curves of the 10×/0.25  NA objective lens [Fig. 6(a)] and FPM system [Fig. 6(b)] slowly decrease to 0.7 at a frequency of 381 and 1055  lp/mm and then precipitously approach zero at the respective cutoff frequencies of 681 and 1487  lp/mm. In contrast, the curves of the 60×/0.95  NA [Fig. 6(c)] and 100×/1.25  NA objective lenses [Fig. 6(d)] decrease rapidly first and then slowly attain zero at the frequencies of 1642 and 2639  lp/mm, respectively.

**Fig. 7:**
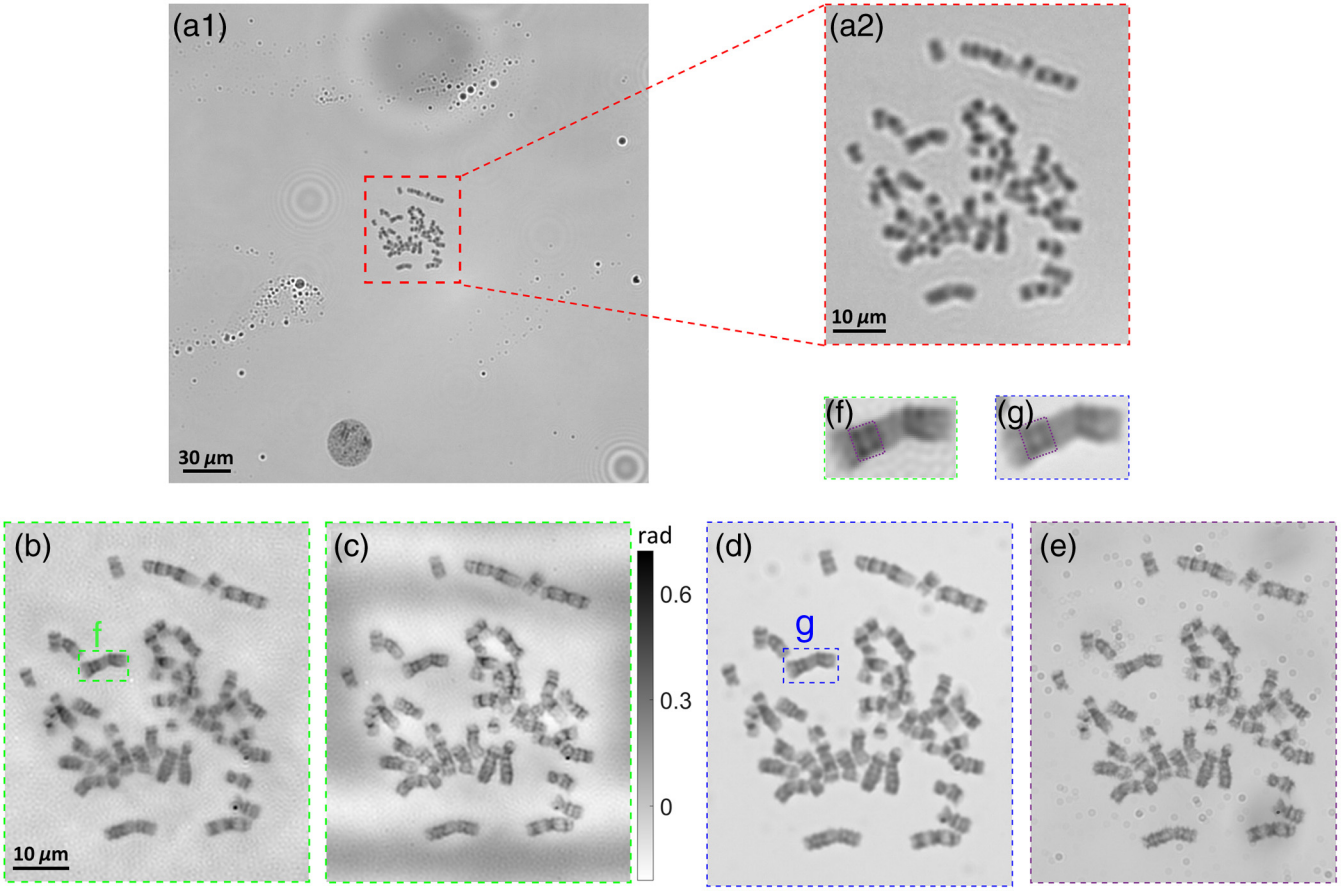
The images of a sample chromosome acquired from the patient’s blood sample, which were captured or reconstructed using (a) 10×/0.25  NA objective lens; (b), (c) FPM technology with 10×/0.25  NA objective lens; (d) 60×/0.95  NA objective lens; and (e) 100×/1.25  NA oil immersion objective lens. (b) and (c) are the reconstructed amplitude and phase images, respectively.

Next, Fig. 7 shows the same analyzable chromosome imaged using FPM technology and different objective lenses. For the result of the 10×/0.25  NA objective lens [Fig. 7(a)], the chromosome bands can be generally recognized, but the pattern details are not recognizable. When using the FPM technology with the 10×/0.25  NA objective [Fig. 7(b)], the quality of the band patterns is vastly enhanced, so the bands are analyzable for the subsequent karyotyping. The reconstructed band patterns are somewhat better than the results of the 60×/0.95  NA objective lens [Fig. 7(d)] but slightly inferior to the results of the 100×/1.25  NA objective lens [Fig. 7(e)].

**Fig. 8:**
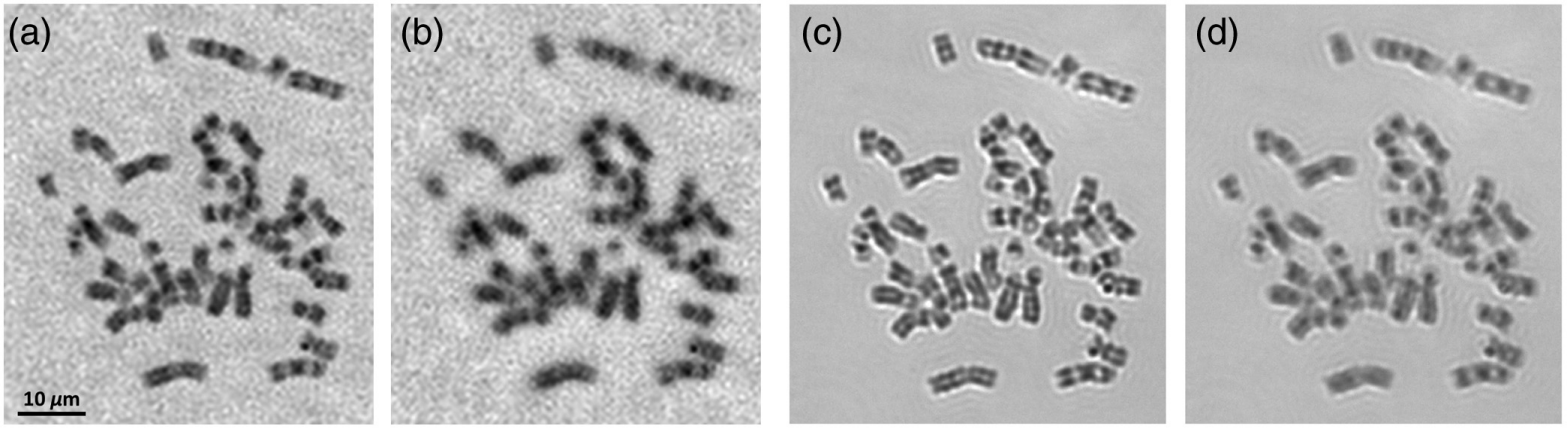
The images of a sample chromosome obtained from the patient’s blood sample. The raw patterns of the chromosome were captured under the 4×/0.1  NA objective lens and reconstructed by FPM technology. The chromosome was placed at the (a) in-focused position and (b) 20  μm away from the in-focused position. The chromosome was captured directly by the 20×/0.4  NA objective lens, which was placed at the (c) in-focused position and (d) 3  μm away from the in-focused position.

**Fig. 9:**
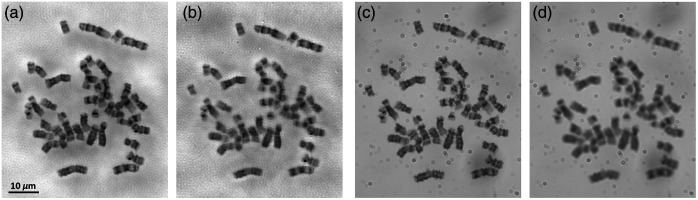
The images of a sample chromosome obtained from the patient’s blood sample. The raw patterns of the chromosome were captured under the 10×/0.25  NA objective lens and reconstructed by FPM technology. The chromosome was placed at the (a) in-focused position and (b) 3.75  μm away from the in-focused position. The chromosome was captured directly by the 100×/1.25  NA objective lens, which was placed at the (c) in-focused position and (d) 2.5  μm away from the in-focused position.

Finally, Figs. 8 and 9 indicate the reconstructed images that were captured at different focusing positions. For Fig. 8(a), the raw patterns used for reconstruction were acquired under the 4×/0.1  NA objective lens at the in-focused position, and the raw patterns of Fig. 8(b) were captured 20  μm away from the in-focused position. Compared with Fig. 8(a), the band patterns in Fig. 8(b) are somewhat deteriorated but still acceptable. Meanwhile, when the chromosomes were directly captured under the 20×/0.4  NA objective lens [Figs. 8(c), 8(d)], the band patterns become somewhat deteriorated when it was placed only 3  μm away from the in-focused position. Similarly, the raw patterns used for the reconstruction of Figs. 9(a) and 9(b) were captured under the 10×/0.25  NA objective lens, which were at in-focused and 3.75  μm away positions, respectively. The band patterns in Fig. 9(b) are generally as sharp as the band patterns in Fig. 9(a). However, when imaging under the 100×/1.25  NA objective lens [Figs. 9(c) and 9(d)], the chromosome bands become fuzzy when the sample is place 2.5  μm away from the focal plane.

## 4 Discussion

In this study, we applied FPM technology on chromosome imaging and thoroughly investigated the band pattern quality depicted on the reconstructed images. To the best of our knowledge, this is the first study that utilizes FPM technology on chromosome imaging, and it has two unique characteristics.

First, although the equivalent NA of our FPM system (raw patterns under the 10×/0.25  NA objective lens) is far <1.25, the band quality of the reconstructed images is close to or even comparable to the results captured by the 100×/1.25  NA objective lens. In the previous research,^22^^,^^23^ it was cautioned that the direct comparison between the equivalent NA of FPM and the native NA of conventional microscopy may not be reasonable. However, our FPM system may better resolve the chromosome samples containing phase information. Meanwhile, we believe this phenomenon can be attributed to two other factors as follows. First, the effective NA of the 10× objective lens is actually higher than its rated value (0.25). As indicated in Fig. 5(a2), the bar patterns are resolvable up to group 9-1, with a half-pitch resolution of 0.98  μm. Accordingly, the computed NA is ∼0.33, which is ∼0.10 higher than 0.25. In this research, the NA of illumination is configured as 0.38; thus the final equivalent NA should be 0.71. This value generally agrees with the measured results [${\mathrm{NA}}_{\mathrm{eq}}$ 0.84, Fig. 5(b)].^9^^,^^14^ Second, the MTF curve is significantly different between the FPM system and equivalent conventional microscope. The FPM curve decreases slowly first and then sharply approaches zero; but the curve of conventional microscope decreases sharply first and then slowly approaches zero. As a result, at a very wide range of frequency (0 to 1000  lp/mm), the FPM microscope can provide better resolving power (i.e., contrast) than the 60×/0.95  NA and 100×/1.25  NA conventional objective lenses, although these two lenses have higher cutoff frequencies. Meanwhile, most chromosome band patterns are bright-dark alternating strips with a spatial frequency of <1000  lp/mm, and the FPM system may provide better contrast on these bands, which explains the comparative quality of the band patterns on the reconstructed images. When the raw patterns were captured under 4× objective lens, the MTF curve of the FPM is similar to the equivalent 20×/0.4  NA objective lens; thus the band pattern qualities are comparable on the images generated by these two systems.

Second, this investigation points out a new strategy to accomplish efficient slide scanning for chromosome karyotyping. For the microscopic chromosome image, we demonstrated that the 10×/0.25  NA FPM system can achieve a comparable result to that of the conventional 100×/1.25  NA objective lens. At the same time, the major advantages of the 10× lens still remain: large FOV and large DOF. This discovery provides a new possible strategy for achieving high-speed chromosome digitization: acquire the raw images under the 10× objective lens and then reconstruct the high-resolution analyzable metaphase chromosomes for the following diagnosis work. In addition, we also demonstrated that the reconstructed images of the 4×/0.1  NA raw patterns achieves a similar quality as compared with the 20×/0.4  NA objective lens, which is adequate for identifying the analyzable cells. Then the efficiency may be further enhanced if the slide is captured and reconstructed first under 4× for the initial screening and then only the selected regions of interest are imaged again under the 10× condition to synthesize high-resolution images. By adjusting the focal length of the tube lens or directly using the available lower magnification objective lens (i.e., 1.25× or 2×), it is also possible to further enlarge the FOV to achieve an even higher scanning speed without any quality degeneration of the final images.

Although the results are encouraging, we recognize that this study has a number of limitations. First, we only used the analyzable metaphase chromosomes acquired from blood samples for feature quality analysis, which is representative but not comprehensive. More types of clinical samples should be investigated, including bone marrow, amniotic fluid, and product of conception.^17^ Second, we did not apply the adaptive wave correct technology^24^ to further enlarge the system DOF. Third, there is still a large potential to enhance the equivalent NA of the FPM system. In this study, the raw patterns were obtained only under 4× and 10× objective lenses, and the illumination NA was only achieved as 0.38. The high-resolving power objective lens (i.e., 20×/0.75  NA) can be used, and the illumination NA can be enhanced to 0.7 by adjusting the LED-sample distance and reconfiguring the illumination pattern on the LED matrix.^14^ Fourth, only the conventional optimization-based reconstruction schemes were adopted in this investigation. The deep learning-based data-driven algorithm is an emerging trend for phase retrieval and pupil function recovery,^25^^–^^27^ but they were not used in this research. Despite these limitations, we believe that this study is highly valuable for the development of a high-throughput chromosome sample digitizer in the future.
